# Comparative and phylogenetic analyses of plastid genomes of the medicinally important genus *Alisma* (Alismataceae)

**DOI:** 10.3389/fpls.2024.1415253

**Published:** 2024-08-20

**Authors:** Zhi-Qiong Lan, Wen Zheng, Alicia Talavera, Ze-Long Nie, Jing Liu, Gabriel Johnson, Xian-Mei Yin, Wen-Qi Zhao, Zong-Yi Zhao, Sara M. Handy, Jun Wen

**Affiliations:** ^1^ State Key Laboratory of Southwestern Chinese Medicine Resources, School of Pharmacy/College of Modern Chinese Medicine Industry, Chengdu University of Traditional Chinese Medicine, Chengdu, China; ^2^ Department of Botany, National Museum of Natural History, Smithsonian Institution, Washington, DC, United States; ^3^ Departamento de Botánica y Fisiología Vegetal, Universidad de Málaga, Málaga, Spain; ^4^ Key Laboratory of Plant Resources Conservation and Utilization, College of Biology and Environmental Sciences, Jishou University, Jishou, China; ^5^ College of Life Science, Sichuan Agricultural University, Ya’an, China; ^6^ Center for Food Safety and Applied Nutrition, Office of Regulatory Science, U.S. Food and Drug Administration, College Park, MD, United States

**Keywords:** *Alisma*, phylogenomics, plastid genome, structural variation, medicinal plant

## Abstract

*Alisma* L. is a medicinally important genus of aquatic and wetland plants consisting of c. 10 recognized species. However, largely due to polyploidy and limited taxon and gene sampling, the phylogenomic relationships of *Alisma* remain challenging. In this study, we sequenced 34 accessions of Alismataceae, including eight of the ten species of *Alisma*, one species of *Echinodorus* and one species of *Luronium*, to perform comparative analyses of plastid genomes and phylogenetic analyses. Comparative analysis of plastid genomes revealed high sequence similarity among species within the genus. Our study analyzed structural changes and variations in the plastomes of *Alisma*, including IR expansion or contraction, and gene duplication or loss. Phylogenetic results suggest that *Alisma* is monophyletic, and constitutes four groups: (1) *A. lanceolatum* and *A. canaliculatum*; (2) the North American clade of *A. subcordatum* and *A. triviale*; (3) *A. wahlenbergii* and *A. gramineum*; and (4) *A. plantago-aquatica* from Eurasia and northern Africa with the eastern Asian *A. orientale* nested within it. Hence the results challenge the recognition of *A. orientale* as a distinct species and raise the possibility of treating it as a synonym of the widespread *A. plantago-aquatica*. The well-known *Alismatis Rhizoma* (Zexie) in Chinese medicine was likely derived from the morphologically variable *Alisma plantago-aquatica* throughout its long history of cultivation in Asia. The plastome phylogenetic results also support the tetraploid *A. lanceolatum* as the likely maternal parent of the hexaploid eastern Asian *A. canaliculatum*.

## 1 Introduction

*Alisma* L. is a medically important genus with about 10 species (Li et al., 2022; Ito and Tanaka, 2023) and is one of the 15 genera of Alismataceae, an ancient aquatic and semi-aquatic group of monocot plants (Les and Tippery, 2013). This genus is widely distributed in temperate and subtropical regions mainly from eastern and South Asia, North America, and Europe to tropical East Africa. Most of the *Alisma* species may be used as food and medicine. For example, *Alisma orientale* and *A. plantago-aquatica* have been utilized in several Asian countries such as China, Japan and Korea (Han et al., 2013) for nearly 2,000 years (Pharmacopoeia of the People’s Republic of China, 2020; The Japanese Pharmacopoeia, 2021). They have been used to treat diseases such as diuresis, oliguria, diabetes, hyperlipidemia, hepatitis and obesity (Liu et al., 2013; Jang et al., 2015). The potential of these two plants’ tubers in cancer treatment had been also explored in one recent research (Jang and Lee, 2021). Furthermore, the extracts of *A. canaliculatum* have been used to treat gastric cancer (Kwon et al., 2021).

Recent phylogenetic studies on Alismataceae employing plastid, mitochondrial and/or nuclear genes have placed *Alisma* as closely related to *Luronium*, *Baldellia* and *Damasonium* (Ross et al., 2016; Lehtonen, 2017; Chen et al., 2022). Of particular interest, Chen et al. (2022) have explored the adaptation to the aquatic environments using Alismataceae as a model with extensive nuclear and organelle data. Based on morphology, geographic distribution, ecology, anatomy and cytology, *Alisma* was divided into two major groups: the *gramineum* group (*A. wahlenbergii*; *A. gramineum*) and the *plantago-aquatica* group (*A. plantago-aquatica*, *A. orientale, A. triviale*) (Hendricks, 1957; Björkqvist, 1968). The groupings are consistent with the taxonomic study on the basis of 38 characters of gross morphology, anatomy and pollen morphology (Zhang and Chen, 1994). However, distinguishing between species within *Alisma* has been challenging due to the similar morphologies among closely related species (Liu et al., 2020).

Several molecular studies have explored the evolutionary history of *Alisma*. An earlier molecular phylogenetic study based on nuclear ribosomal internal transcribed spacer (nrITS), plastid DNA (*trnL*), and RAPD markers (Jacobson and Hedrén, 2007) supported the two major diploid groups (i.e. the *plantago-aquatica* group and the *gramineum* group), but the positions of the polyploid *Alisma* species (*A. canaliculatum*, *A. lanceolatum*, *A. triviale* and *A. rariflorum*) have remained uncertain. Focusing on the family-level phylogenetic analyses, Chen et al. (2012) sampled only four species of *Alisma*: *A. canaliculatum, A. gramineum, A. nanum* and *A. plantago-aquatica* based on nrITS and plastid DNA (*matK, psbA* and *rbcL*) data. Some topological inconsistencies were observed, with *A. gramineum* being resolved as sister to the other three species in their nrITS tree, but the plastid data supported two clades with the *A. gramineum* - *A. nanum* clade being sister to the *A*. *plantago-aquatica* - A*. canaliculatum* clade.

Recently Ito and Tanaka (2023) performed a phylogenetic analysis of *Alisma* based on several DNA fragments including nuclear (nrITS and *phyA*) and chloroplast (*matK*, *ndhF*, *psbA-trnH* and *rbcL*) markers. The study focused on Asian species, sampling six putative species and two varieties particularly from Japan and Europe. The result suggested that *A. canaliculatum* from eastern Asia and *A*. *rariforum* endemic to Japan are sister to each other. *A. orientale* collected from China, Myanmar and Vietnam was shown to be nested within *A*. *plantago-aquatica*. Ito and Tanaka (2023) suggested that *A. orientale* was likely derived through parapatric speciation in the mountainous regions at the southern edge of the distribution of *A*. *plantago-aquatica*.

Due to limited taxon sampling, molecular markers, and small morphological differences between species, as well as the polyploidy of some species, the phylogenetic position of species of *Alisma* remains uncertain and controversial, such as the low support and ambiguous affinities of the three species of *A. plantago-aquatica*, *A. orientale* and *A. canaliculatum* (Ito and Tanaka, 2023). Therefore, the phylogenetic relationships of *Alisma* still need further exploration. Compared with plastid gene fragments, plastid genomes (plastomes) contain more informative information. The highly conserved structure of plastid genome is suitable for inferring the evolutionary relationships of higher taxa (Givnish et al., 2018). Plastome sequences have been extensively used to articulate phylogenetic relationships of many plant genera (Lv et al., 2022; Su et al., 2022; Xu et al., 2022; Hu et al., 2023). However, whole plastome sequence data, which could be used to develop novel genetic markers for determining infrageneric relationships, are still lacking for most species of *Alisma*.

In this study, we sequenced and performed comparative analyses of the plastid genomes of *Alisma*. The specific aims of this study were to: (1) investigate the structural variation of plastome genomes in *Alisma* at both the population and species levels, and infer the plastome structural evolution of *Alisma*; (2) identify the most variable regions of these plastomes as reference genes for future species identification; and (3) uncover the phylogenetic relationships within *Alisma*. We hope the phylogenetic study of *Alisma* using whole plastomes will lead to a better phylogenetic framework and improve resource conservation and the downstream application of medicinal resources, which are important for the modern utilization of traditional natural herbs.

## 2 Materials and methods

### 2.1 Plant material, DNA extraction and sequencing

A total of 34 accessions, including eight recognized species of *Alisma*, one species of *Echinodorus* Rich. and one species of *Luronium* Raf. of Alismataceae were sampled. All voucher specimens have been deposited in the United States National Herbarium (US), the KHD Herbarium of the Denver Botanic Gardens (KHD), the University of Göttingen Herbarium (GOET), and the Chinese Medicine Herbarium of Chengdu University of Chinese Medicine (CDCM). The voucher information is presented in Supplementary **Table 1**. Additionally, chloroplast genome data from six accessions, representing *Sagittaria* (NC_067603, NC_044119, NC_029815) and *Caldesia* (NC_045925, NC_045926) of Alismataceae, and *Butomus* (NC_051949) of Butomaceae (Alismatales), were obtained from the NCBI (Luo et al., 2016; Liang et al., 2019; Mwanzia et al., 2019; Yang and Liu, 2019) (**Supplementary Table 1**).

**Table 1:** Plastome features of 31 species of *Alisma* presented in this study.

Species	Complete		LSC		SSC		IR		Number of genes	CDS	tRNA	rRNA	accession number
	Length(bp)	GC(%)	Length(bp)	GC(%)	Length(bp)	GC(%)	Length(bp)	GC(%)					
*A. canaliculatum*	159,629	36.00	89,404	33.64	19,455	29.36	25,385	42.70	113	79	30	4	OR999289
*A. canaliculatum*	159,471	36.00	89,248	33.64	19,497	29.31	25,363	42.73	113	79	30	4	OR999290
*A. gramineum*	159,925	36.00	89,586	33.70	19,789	29.18	25,275	42.75	113	79	30	4	OR999291
*A. lanceolatum*	159,543	36.00	89,588	33.66	19,535	29.26	25,210	42.77	113	79	30	4	OR999292
*A. lanceolatum*	159,797	36.00	89,529	33.66	19,494	29.26	25,387	42.72	113	79	30	4	OR999293
*A. lanceolatum*	159,666	36.00	89,378	33.64	19,472	29.28	25,408	42.72	113	79	30	4	OR999294
*A. lanceolatum*	159,795	36.00	89,501	33.65	19,488	29.27	25,403	42.72	113	79	30	4	OR999295
*A. lanceolatum*	159,599	36.00	89,597	33.66	19,528	29.25	25,237	42.76	113	79	30	4	OR999296
*A. lanceolatum*	159,795	36.00	89,501	33.65	19,488	29.27	25,403	42.72	113	79	30	4	OR999297
*A. lanceolatum*	159,801	36.00	89,502	33.65	19,493	29.27	25,403	42.72	113	79	30	4	OR999298
*A. orientale*	159,861	36.00	89,565	33.69	19,786	29.17	25,255	42.78	113	79	30	4	OR773541
*A. orientale*	159,853	36.00	89,523	33.69	19,770	29.20	25,280	42.75	113	79	30	4	OR999299
*A. plantago-aquatica*	159,889	36.00	89,590	33.68	19,749	29.20	25,275	42.76	113	79	30	4	OR999300
*A. plantago-aquatica*	159,910	36.00	89,547	33.71	19,773	29.17	25,295	42.73	113	79	30	4	OR999301
*A. plantago-aquatica*	159,902	36.00	89,582	33.69	19,770	29.18	25,275	42.76	113	79	30	4	OR999302
*A. plantago-aquatica*	159,904	36.00	89,565	33.69	19,773	29.17	25,283	42.76	113	79	30	4	OR999303
*A. plantago-aquatica*	159,866	36.00	89,532	33.70	19,774	29.17	25,280	42.75	113	79	30	4	OR999304
*A. plantago-aquatica*	159,871	36.00	89,541	33.69	19,770	29.18	25,280	42.75	113	79	30	4	OR999305
*A. plantago-aquatica*	159,859	36.00	89,526	33.70	19,773	29.17	25,280	42.75	113	79	30	4	OR999306
*A. plantago-aquatica*	159,904	36.00	89,565	33.69	19,773	29.17	25,283	42.76	113	79	30	4	OR999307
*A. plantago-aquatica*	159,904	36.00	89,565	33.69	19,773	29.17	25,283	42.76	113	79	30	4	OR999308
*A. plantago-aquatica*	159,893	36.00	89,571	33.69	19,772	29.16	25,275	42.76	113	79	30	4	OR999309
*A. plantago-aquatica*	159,686	36.00	89,424	33.67	19,752	29.21	25,255	42.78	113	79	30	4	OR999315
*A. subcordatum*	159,728	36.00	89,467	33.67	19,751	29.18	25,255	42.80	113	79	30	4	OR999311
*A. subcordatum*	159,587	36.00	89,323	33.67	19,754	29.17	25,255	42.80	113	79	30	4	OR999310
*A. subcordatum*	160,180	36.00	89,465	33.67	19,277	29.14	25,277	42.59	113	79	30	4	OR773542
*A. triviale*	159,726	36.00	89,465	33.67	19,751	29.18	25,255	42.80	113	79	30	4	OR999312
*A. triviale*	159,731	36.00	89,470	33.67	19,751	29.18	25,255	42.80	113	79	30	4	OR999313
*A. triviale*	159,734	36.00	89,471	33.67	19,753	29.18	25,255	42.80	113	79	30	4	OR999314
*A. triviale*	159,727	36.00	89,466	33.66	19,751	29.19	25,255	42.80	113	79	30	4	OR773543
*A. wahlenbergii*	159,886	36.00	89,588	33.69	19,736	29.19	25,281	42.75	113	79	30	4	OR999316

Total genomic DNAs were isolated from silica-gel dried leaves or herbarium specimens using a modified SDS method (Dellaporta et al., 1983; Johnson et al., 2023). DNA concentration was determined with a Qubit 4.0 fluorometer (Thermo Fisher Scientific) using a high sensitivity dsDNA kit while integrity and fragment size were evaluated with agarose gel electrophoresis (1% agarose SeaKem LE (Lonza Group Ltd. Basal, Switzerland)). DNA libraries were prepared using a KAPA HyperPrep Kit (Hoffmann-La Roche, Basel, Switzerland) and then sequenced with an Illumina Nova-Seq 6000 platform (paired-end, 2x150bp) at Novogene, Sacramento, CA, USA.

### 2.2 Raw data processing and sequence assembly

The raw reads were trimmed using Trimmomatic v.0.39 (Bolger et al., 2014). We used FastQC v.0.12.1 (http://www.bioinformatics.babraham.ac.uk/projects/fastqc/) to check the quality of the sequences. For the chloroplast genomes assembly, we employed GetOrganelle v.1.7.7 (Jin et al., 2020) based on trimmed paired-end reads. Circular genomes were generated for 27 taxa. For the seven samples which failed to be assembled by the one step from GetOrganelle due to likely low sequence coverage of some parts, a manual assembly method using closely related references was applied (Zhang et al., 2015; Liu et al., 2019).

For the plastid genomes, *de novo* assembly graphs were visualized and edited using Bandage v.0.8.1, and a whole or nearly whole circular plastid genome was generated (Wick et al., 2015). Using the plastid genome of *A. orientale* (OR522698) downloaded from NCBI (National Centre for Biotechnology Information, https://www.ncbi.nlm.nih.gov/) as a reference, all the genes were predicted using CPGAVAS v.2.0 (Liu et al., 2012). Subsequently, the annotation of the new sequences was then manually checked using Geneious v.11.0.18 (Kearse et al., 2012). All the plastid genomes from *Alisma*, *Echinodorus* and *Luronium* specie, were newly obtained in the present study, and submitted to the NCBI after being checked without error. The accession numbers of all samples are shown in **Supplementary Table 1**. These raw and annotated sequences have also been added to the United States Food and Drug Administration’s GenomeTrakrCP database and the related Bioproject (PRJNA325670, Zhang et al., 2017a). Circular genome visualization was illustrated using the online tool Organellar Genome Draw (OGDRAW) v.1.3.1 (https://chlorobox.mpimpgolm.mpg.de/OGDraw.html) (Greiner et al., 2019).

### 2.3 Genome structure and repeat sequence analysis

Geneious was utilized to count the number of genes, types and other information of all samples. The LSC, SSC, and IR regions of each chloroplast genome sequence were also extracted, and the AT and GC contents were counted using BioEdit (Alzohairy, 2011).

REPuter software (Kurtz et al., 2001) was used to identify forward (F), reverse (R), palindrome (P), and complementary (C) repeats in the resultant plastomes with the setting of a minimum repeat size of 30 bp and a maximum of 1000 bp, with sequence identities greater than 90% (Hamming Distance of 3). Simple sequence repeats (SSRs) were predicted using MISA (Beier et al., 2017) by setting the minimum number of repeat units to 10, 5, 4, 3, 3, and 3 for the mono-, di-, tri-, tetra-, penta- and hexa-nucleotides. The maximum length of the sequence between two SSRs to register as a compound SSR was set to 0 bp.

### 2.4 Comparative genomic analyses

To explore the expansion and contraction of IR regions of *Alisma*, comparison of boundaries between IRs and single copy regions was performed in CPJSdraw v.1.0.0 (Li et al., 2023). The mVISTA (https://genome.lbl.gov/vista/mvista) (Mayor et al., 2000) was used to assess the similarity among the plastid genomes, and the default parameters were utilized to align the cp genomes in Shuffle-LAGAN mode with *A. orientale* (OR522698) as the reference. Common sequences of coding regions and intergenic spacer regions (IGS) from 31 samples of *Alisma* were extracted.

Multiple alignments of sequences were performed using MAFFT v.7.48 with default settings (Katoh et al., 2019). Sliding window analysis of nucleotide variability in the cp genome was conducted using DnaSP v.6.11 (Rozas et al., 2017). The step size was set to 200 bp, with a 600 bp window length.

### 2.5 Phylogenetic analyses

To explore the phylogenetic relationships in *Alisma*, we included not only the newly sequenced samples of this genus, but also eight species from other genera within Alismataceae (*Caldesia, Echinodorus, Luronium* and *Sagittaria*). *Botomus umbellatus* (Butomaceae) of Alismatales (NC_051949) downloaded from Genbank was used as an outgroup. The matrix for phylogenomic analyses consisted of complete aligned plastid genomes, and the global alignment was done using MAFFT (Katoh et al., 2019). Poorly aligned regions were then removed using trimAl v1.2 (Capella-Gutiérrez et al., 2009). The final matrix had a total length of 159,457 bp for a total of 40 individuals.

A maximum likelihood (ML) analysis of the complete plastome data was implemented using IQ-TREE v.1.6 (Nguyen et al., 2015) with support values estimated by 10,000 ultrafast bootstrap replicates. The best fit model GTR+I+G model of molecular evolution was used, and the best-fit partitioning schemes and models estimated by ModelFinder (Kalyaanamoorthy et al., 2017). The Bayesian Inference (BI) using MrBayes v.3.2.7 (Ronquist et al., 2012), and the Markov Chain Monte Carlo (MCMC) algorithm was calculated for 1,000,000 generations with a sampling tree every 1000 generations.

## 3 Results

### 3.1 Organization of the plastid genomes of species in *Alisma*

The length of complete plastomes of *Alisma* ranged from 159,471 bp (*A. canaliculatum*) to 160,180 bp (*A. subcordatum*). All of the plastomes displayed a typical quadripartite architecture that contained a large single copy (LSC: 89,248–89,597 bp) and a small single copy (SSC: 19,277–19,789 bp) separated by two copies of an inverted repeat (IR: 25,210–25,408 bp). The total GC content is 36.00%, and the IR regions had the highest GC content (42.70–42.80%) followed by those in the LSC (33.64–33.71%) and SSC (29.14–29.36%) regions (**Table 1**).

Each plastome encoded 113 unique genes, including 79 protein-coding, 30 tRNA and 4 rRNA genes. Amongst them, 17 genes were duplicated in the IR regions, comprising six protein-coding genes (*rps7, rps12, rpl2, rpl23, ndhB* and *ycf2*), four rRNA genes (*rrn4.5, rrn23, rrn5* and *rrn16*), and seven tRNA genes (*trnA-UGC, trnI-CAU, trnI-GAU, trnL-CAA, trnN-GUU, trnV-GAC* and *trnR-ACG*). 17 intron-containing genes were detected, including ten protein-coding genes (*atpF, ndhA, ndhB, rpl2, rpl16, rps16, rps12, clpP, rpoC1* and *ycf3*) and seven tRNA genes (*trnH-GUG, trnG-UCC, trnA-UGC, trnI-GAU, trnV-UAC, trnK-UUU* and *trnL-UAA*). Of the 17 genes, three (*clpP, rps12* and *ycf3*) harbored two introns and the other 14 contained only one intron (**Table 2**).

**Table 2:** Genes present in the plastid genomes of *Alisma* species.

Category for genes	Group of genes	Name of genes
Self-replication	Ribosomal RNA genes	*rrn16* ^a^ *,rrn23* ^a^ *,rrn4.5* ^a^ *,rrn5* ^a^
	Transfer RNA genes	*trnH-GUG* ^b^ *,trnQ-UUG,trnS-GCU,trnG-UCC* ^b^ *,trnR-UCU,trnC-GCA,trnD-GUC,trnY-GUA,trnE-UUC,trnT-GGU,trmM-CAU,trnS-UGA,trnfM-CAU,trnS-GGA,trnT-UGU,trnL-UAA* ^b^ *,trnF-GAA,trnV-UAC* ^b^ *,trnW-CCA,trnP-UGG,trnP-GGG,trnL-CAU* ^a^ *,trnL-CAA* ^a^ *,trnV-GAC* ^a^ *,trnI-GAU* ^ab^ *,trnA-UGC* ^ab^ *,trnR-ACG* ^a^ *,trnN-GUU,trnL-UAG,trnN-GUU,trnH-CAU,trnK-UUU* ^b^
	Ribosome large subunit gene	*rpl2* ^ab^ *,rpl14,rpl16* ^b^ *,rpl20,rpl22,rpl23* ^a^ *,rpl32,rpl33,rpl36*
	Ribosome small subunit gene	*rps2,rps3,rps4,rps7* ^a^ *,rps8,rpsl1,rps12* ^ab^ *,rps14,rps15,rps16* ^b^ *,rps18,rps19*
	DNA-dependent RNA polymerase gene	*rpoA,rpoB,rpoC1* ^b^ *,rpoC2*
	Translation initiation factor	*infA*
Genes for photosynthesis	Subunits of photosystem I	*psaA,psaB,psaC,psal,psaJ*
	Subunits of photosystem II	*psbA,psbB,psbC,psbD,psbE,psbF,psbH,psbI,psbJ,psbK,psbL,psbM,psbN,psbT,psbZ*
	Cytochrome subunits	*petA,petB* ^b^ *,petD* ^b^ *,petG,petL,petN*
	ATP synthase subunit	*atpA,atpB,atpE,atpF* ^b^ *,atpH,atpI*
	ATP-dependent protease subunit	*clpP* ^b^
	Rubisco large subunit	*rbcL*
	NADH dehydrogenase subunit	*ndhA* ^b^ *,ndhB* ^ab^ *,ndhC,ndhD,ndhE,ndhF,ndhG,ndhH,ndhl,ndhJ,ndhK*
Other genes	Mature enzyme genes	*matK*
	Envelope protein gene	*cemA*
	Subunit of Acetyl-carboxylase	*accD*
	C-type cytochrome synthesis gene	*ccsA*
Genes of unknown function	Open reading frames (ORF, ycf)	*ycfl,ycf2,ycf3* ^b^ *,ycf4*

The “a” label after gene names reflects genes located in IR regions. Intron containing gene is indicated by “b”.

### 3.2 SSRs and long repeat sequence

**Figure 1:**
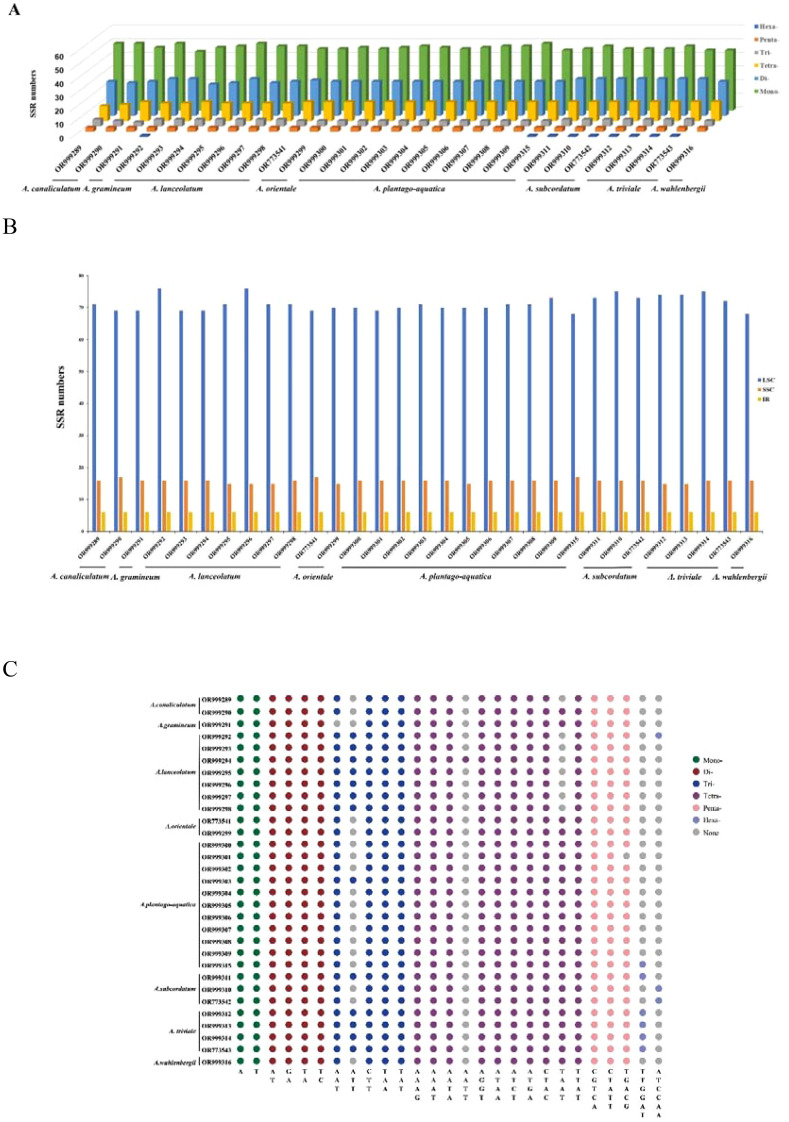
Analysis of simple sequence repeats (SSRs) in the plastid genomes. **(A)** Number of different SSR types detected in the genomes; **(B)** number of SSRs identified in LSC, SSC, and IR regions; **(C)** SSR motifs in different repeat types.

The number of SSRs in each plastome of *Alisma* ranged from 90 (*A. triviale*) to 98 (*A. lanceolatum*). The majority of the SSRs were mononucleotides (49.62%), followed by tetranucleotides (27.41%), dinucleotides (14.66%), trinucleotides (4.78%) and pentanucleotides (3.22%). The smallest number of SSRs were hexanucleotides (0.31%). The mononucleotide SSRs were composed of A/T mofits and most of the dinucleotide ones were composed of AT/TA. SSR was mainly located in the LSC region (76.51%), followed by SSC (17.05%), and then IR (6.44%) (**Figure 1**).

**Figure 2:**
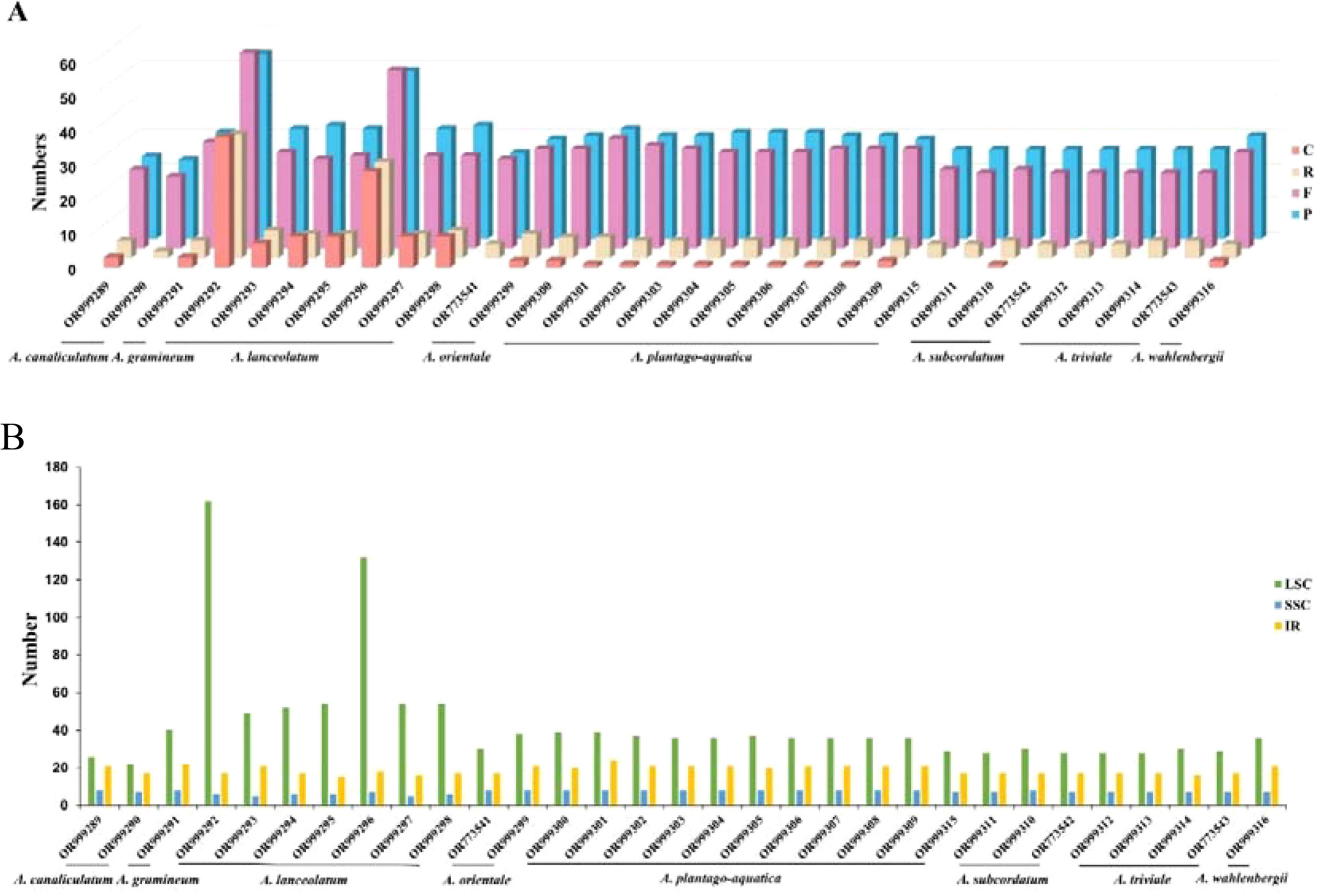
Repeat sequences in the plastid genomes of *Alisma*. **(A)** Number of Repeat sequences types detected in the genomes; **(B)** number of Repeat sequences identified in LSC, SSC, and IR regions.

A total of 2157 long repeat sequences with lengths greater than 30 bp, including 871 (40.38%) forward repeats, 939 (43.53%) palindromic repeats, 132 (6.12%) complementary repeats and 215 (9.97%) reverse repeats, were detected in 31 samples from *Alisma.* The number of repeats for each taxon varied from 46 to 185 and had lengths that ranged from 30 to 55 bp. Most of the long repeats were located in LCS region (62.45%), followed by IR (27.17%) and SSC (10.38%) (**Figure 2**).

### 3.3 Expansion and contraction of the IR region

**Figure 3:**
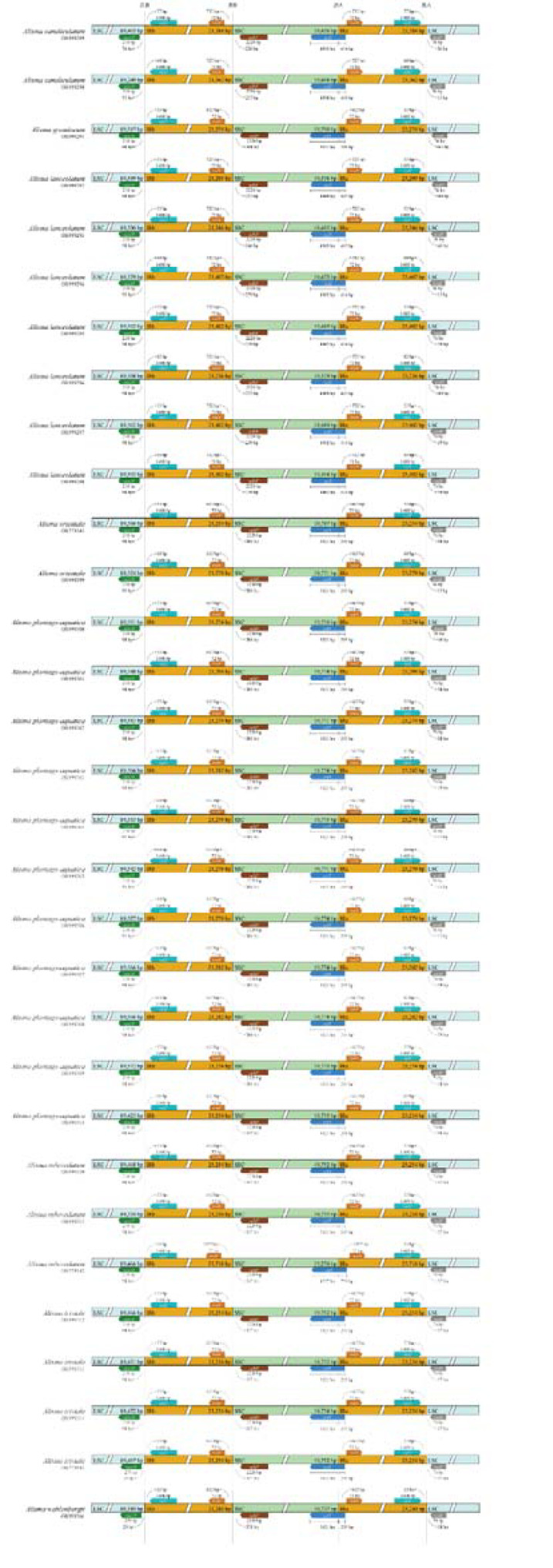
Comparison of junctions of LSC, SSC, and IR regions in the plastid genomes of *Alisma.*.

Four boundaries of the plastid genomes, namely JLB (LSC-IRb), JSB (SSC-IRb), JSA (SSC-IRa), and JLA (LSC-IRa) showed remarkable stability among the species in *Alisma*. The IR/SC junctions of the plastid genomes mainly contained five genes (*rpl2, rps19, ycf1, ndhF* and *trnH*). The rps19 gene was completely present within the LSC, at a distance of 29-98 bp to the JLB junction. For the JLA junction, the *trnH* gene was located in the LSC region 13-64 bp away from the junction, whereas the *rpl2* gene was located in the IRa region 55-82 bp away from the junction. We only detected differences in distance to the JLA junction. The SSC/IRa boundary was located within the *ycf1* gene and had lengths ranging from 4,557 bp to 5,021 bp in the SSC and of 295 bp or 759 bp in the Ira. In total, expansion of IRa boundaries in *A. canaliculatum* and *A. lanceolatum* compared to other species. Genetic variation is somewhat intraspecific conserved and interspecific differences are relatively pronounced in *Alisma* (**Figure 3**).

### 3.4 Hypervariable regions of the plastid genomes

**Figure 4:**
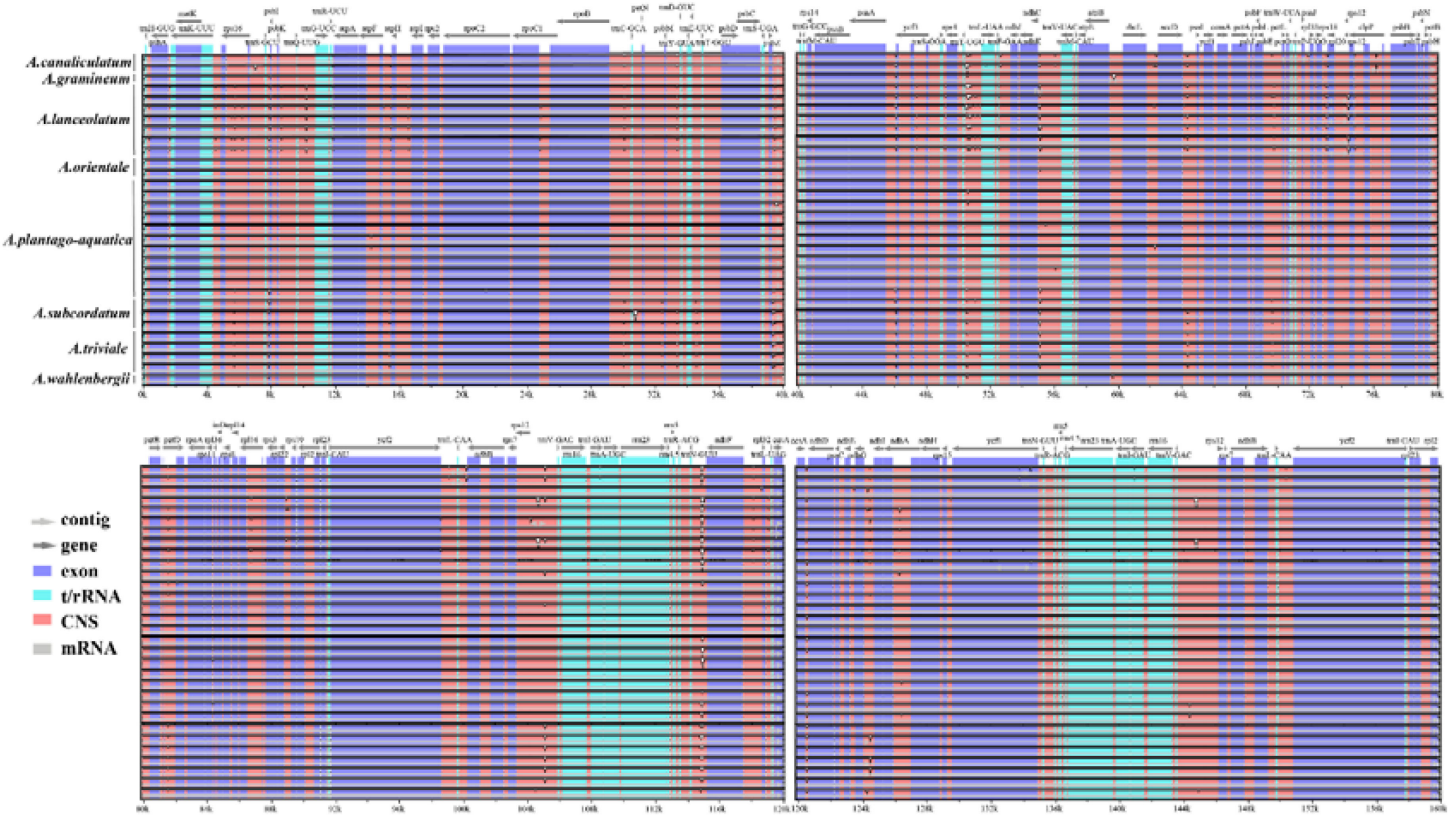
Sequence alignment of the 31 plastid genomes performed using the mVISTA program with *Alisma orientale* (OR522698) as a reference.

The complete plastid genomes from *Alisma* were compared using mVISTA with the *A. orientale* (OR522698) genome as reference. Comparative analysis showed that the plastid genomes were evolutionarily conserved with similar structures and gene orders. IR regions were found to be more conserved than the single copy regions, so were genic regions, coding regions, and exons compared with intergenic regions, non-coding and introns. The regions of 4-12 kb, 46-56 kb, 64-66 kb, 104-106 kb, 114-116 kb, and 124-128 kb have relatively large variations (**Figure 4**).

**Figure 5:**
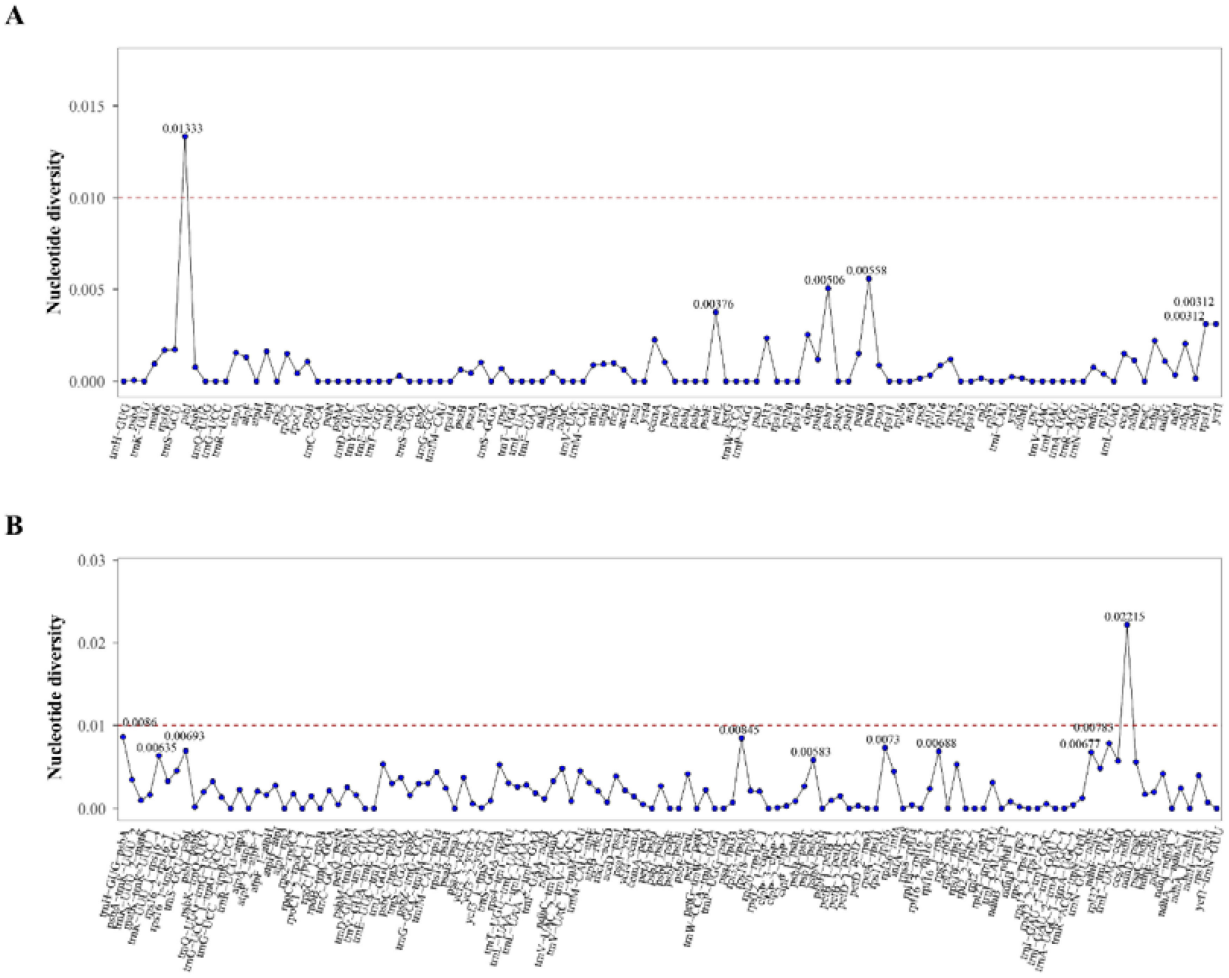
Sliding window analysis of *Alisma* species. **(A)** Nucleotide diversity values of common genes within the chloroplast genomes; **(B)** Nucleotide diversity values of intergenic spacers (IGS) within the chloroplast genomes.

A sliding window analysis was used to estimate the level of variation across regions in coding and non-coding regions. Nucleotide diversity (Pi) values ranged from 0.00000 to 0.01333 for coding regions and 0.00000 to 0.02215 for non-coding regions. All highly divergent sequences were restricted to the single copy (SC) regions, with the highest peak occurring in the SSC region. Among these regions, one coding regions with Pi values exceeding 0.01 and one regions in IGS regions with Pi values surpassing 0.02 were identified as significant hotspot regions, of which one was *psbI* gene, and the remaining one was intergenic spacer of *ccsA-ndhD* (**Figure 5**).

### 3.5 Phylogenetic inference

**Figure 6:**
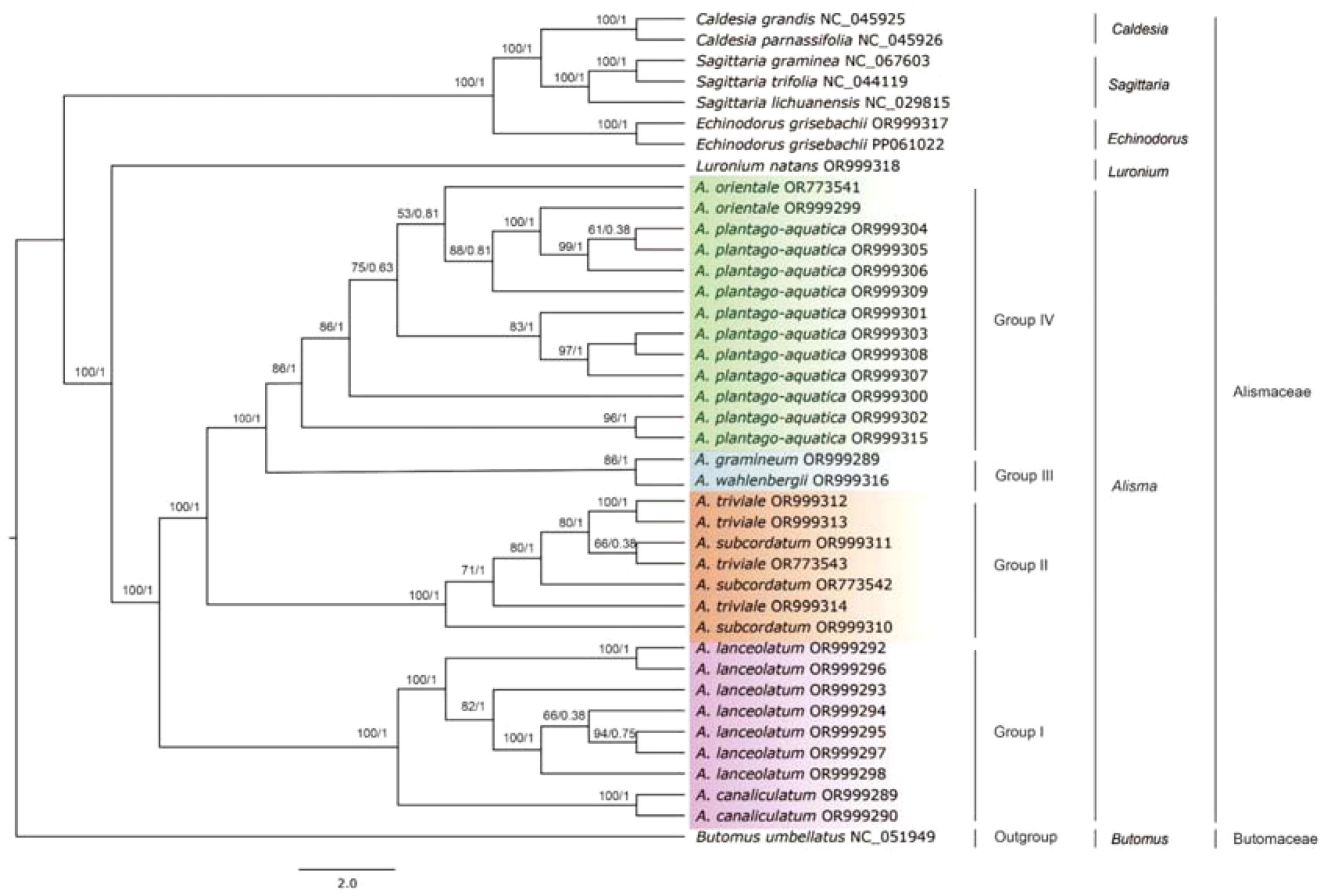
Plastid phylogeny of *Alisma* using Maximum Likelihood (ML) and Bayesian Inference (BI) methods based on the whole plastome sequences. Likelihood bootstrap support values (BS) and Bayesian posterior probability values (PP) are shown next to the nodes.

The plastid genome topologies of *Alisma* based on ML and BI analyses were consistent (**Figure 6**). The monophyly of *Alisma* was strongly supported (100%ML BS, 1.0 PP). The whole plastid genome data provided strong support for the position of *A. canaliculatum* (100% ML BS, 1 PP), *A. lanceolatum* (100% ML BS, 1 PP), *A. wahlenbergii* (86% ML BS, 1 PP) and *A. gramineum* (86% ML BS, 1 PP). The eight *Alisma* species sampled were grouped into four clades. Group I consists of *A. canaliculatum* and *A. lanceolatum*, which diverged first. Group II is composed of two species, *A. subcordatum* and *A. triviale*, both from North America. Groups III and IV were sister to each other, and each clade is composed of two species. Group III includes *A. gramineum* and *A. wahlenbergii*, which are very similar in morphology. It should be noted that the two species *A. plantago-aquatica* and *A. orientale* in Group IV are largely distributed in Eurasia and they cannot be clearly delineated. Furthermore, the two North American species, *A. subcordatum* and *A. triviale* in Group II are intermixed, and each did not form a clade (**Figure 6**).

## 4 Discussion

### 4.1 Gene content and structural variations of *Alisma* plastomes

Plastome structure and gene content are generally considered to be conserved in flowering plants, but vary in length from 120 kb to 160 kb and consist of 100-120 unique genes (Bai et al., 2021). In this study, 113 genes were annotated across the genomes of the species in *Alisma* and one specie in *Luronium*, including 79 protein-coding genes, 30 tRNA genes, and four rRNA genes that showed good conservatism across species in this genus. The length of complete plastid genomes of *Alisma* ranged from 159,471 bp in *A. canaliculatum* to 160,180 bp in *A. subcordatum*, which are clearly shorter than *Sagittaria* and *Caldesia* in Alismataceae (Liang et al., 2019; Mwanzia et al., 2019). However, the length of the plastid sequences of the *Alisma* species is similar to that of Hydrocharitaceae and Butomaceae, which are close relatives of Alismataceae in Alismatales. In addition to the obvious difference in length, the genera of Alismataceae also differed greatly in the type, number and length of *ycf* genes. For example, *Caldesia parnassifolia* (NC_045926) did not contain *ycf1* gene; *Sagittaria graminea* (NC_067603) contained two *ycf1* genes; and the 32 samples from *Alisma* and *Luronium* sequenced in the present study contained only one *ycf1* gene. In addition to the Alismataceae, the Alismatales contain other species with deletions of *ycf1* genes, including the Hydrocharitaceae, Butomaceae, and Tofieldiaceae. In general, although *ycf1* is a highly variable gene that is often used as a DNA barcode in phylogenetic analyses (Dong et al., 2015; Zhao et al., 2020), *ycf1* remains highly conserved among species within *Alisma* as this study shows.

SSRs have the lowest number of hexanucleotides, with only one hexanucleotide found in nine samples from three *Alisma* species, including *A. triviale* (OR999314, OR773543, OR999312, OR999313)*, A. subcordatum* (OR999311, OR773542, OR999310), *A. lanceolatum* (OR999292) and *A. plantago-aquatica* (OR999315). No hexanucleotides were found in the other samples. Notably, *A. triviale* and *A. subcordatum* are two species from North America. They had slightly more SSRs than other species of the genus, and both species contained hexanucleotides SSRs. Meanwhile, most of the SSRs are located in the LSC and SSC, revealing conservatism of the IR regions, which was also supported by the distributions of the hypervariable regions (HVR). As SSRs are highly reliable, reproducible, and highly polymorphic, analysis of the SSR loci in the plastid genomes of *Alisma* has the potential for future population genetic studies, genetic diversity and species identification (Raza et al., 2019; Tang et al., 2021).

Among the four long repetitive sequences, forward repeats, palindromic repeats, complementary repeats, and reverse repeats, we identified the lowest number of complementary repeats. In *A. triviale* (OR999314, OR773543, OR999312, OR999313), *A. subcordatum* (OR999311, OR773542), *A. plantago-aquatica* (OR999315), *A. orientale* (OR773541) and *A. canaliculatum* (OR999290) in which no complementary repeat sequences were found. However, 38 and 28 complementary repeats were respectively found in two samples of *A. lanceolatum* (OR999292, OR999296). The number of complementary repeats found in other samples of *A. lanceolatum* was also higher than that of other species. Long repetitive sequences may promote variation and rearrangement in the plastid genomes, thus they play an important role in plant evolution (Wang et al., 2008). The plastome variation in *A. lanceolatum* may be further explored with a broader populational sampling.

Although it is generally accepted that the plastid genome is quite conserved in terms of structural organization and gene arrangement, expansion and contraction of the IR region at the boundary with the LSC and SSC regions are extremely common in chloroplast genomes (Wang et al., 2018; Ye et al., 2018). These changes are closely related to the differences in chloroplast genome length between taxa at the taxonomic hierarchy above the genus level (Stamatakis, 2014). In this study, by comparing the boundary regions of 31 *Alisma* plastid genomes, the IR boundaries of both *A. canaliculatum* and *A. lanceolatum* were found to be somewhat expanded, but the size and distribution of genes in the boundary region of the taxon remained conservative. It is also evident that the differences among the four boundary regions combined could not clearly and accurately reflect the inter- and intraspecific differences in *Alisma*. This further suggests that the developmental process of *Alisma* also relies on genetic changes in other regions.

The result of the global visualization showed that the *Alisma* plastid genomes had considerable similarity, little interspecific variation, and are developmentally conserved, with coding regions showing stronger conservation than non-coding regions, and the IR region being more conserved than the two single-copy regions. *A. canaliculatum* and *A. lanceolatum* had more variable sites compared to the reference sequence (*A. orientale* OR522698). In addition, *A. subcordatum* and *A. triviale* from North America also showed greater variation than others. Combined with the analysis of nucleotide polymorphisms, the degree of variation in the non-coding regions of the chloroplast genome is generally higher than that in the coding regions, which is consistent with the results of most plant studies (Zhao et al., 2020; Su et al., 2022). Despite the typically stable nucleotide content and highly conserved gene structure of chloroplast genomes, mutation hotspots can still occur. Among the protein-coding genes, the *psbI* gene (pi = 0.01333) had a relatively high level of polymorphism, the intergenic spacer of *ccsA-ndhD* had the highest polymorphism (pi = 0.02215). They can be considered as a molecular marker for *Alisma* systematic, genealogical and biogeographic studies. However, compared to other genera, such as *Gaultheria* (Xu et al., 2022), *Alisma* has fewer loci with significant nucleotide variants and low nucleotide diversity values. Alismataceae is one of the oldest lineages within monocots (Chen et al., 2012). However, the low genetic diversity in *Alisma* suggests that while it has an ancient origin, it diverged relatively recently. In addition, the high hybridization affinity among species of *Alisma* (Jacobson and Hedrén, 2007) may also be responsible for the relatively weak reproductive isolation among *Alisma* species.

### 4.2 Plastid phylogenomics of *Alisma*

According to our ML and BI trees using the complete plastomes (**Figure 6**), *Alisma* is divided into four groups (I, II, III, and IV) (100% ML BS, 1 PP). Group I, including *A. lanceolatum* and *A. canaliculatum*, is sister to all other taxa of the genus; Group II consists of two New World species (*A. subcordatum* and *A. triviale*) and is sister to the clade of Group III (*A. wahlenbergii* and *A. gramineum*) and Group IV (*A. plantago-aquatica* and *A. oriental*e).

Group I: Two Eurasian species (*A. lanceolatum* and *A. canaliculatum*): Our plastome phylogeny showed that two polyploid species *A. lanceolatum* (2*n* = 28) and *A. canaliculatum* (2*n* = 42) constitute one clade (100% ML BS, 1PP). However, the phylogenetic analyses result based on the nuclear ITS and RAPD data (Jacobson and Hedrén, 2007) did not place these two species together. *A. lanceolatum* from temperate Europe, western Asia and North Africa was unresolved in the RAPD tree, while it was sister to the clade of the remaining *Alisma* species in the ITS tree. Jacobson and Hedrén (2007) suggested an ancient allotetraploid origin of *Alisma lanceolatum*. The placement of the hexaploid eastern Asian *A. canaliculatum* as the sister to the widespread *A. lanceolatum* suggests that *A. lanceolatum* may have served as the maternal parental species for *A.* canaliculatum. Our result of the IR boundary analysis and sequence variation analysis also showed that *A. canaliculatum* and *A. lanceolatum* differed to a greater extent from other species within the genus and to a lesser extent between the two species.

Björkqvist (1968) found that *A. canaliculatum* and *A. lanceolatum* were both very uniform and well distinguished in many characteristics from other taxa of the genus, and are most similar to each other vegetatively. Yet the leaf shape of the two species was usually different. The two species have aerial leaves that are lanceolate to broadly lanceolate and have cuneate leaf bases. But *A. lanceolatum* has thin and translucent lateral pericarps, substraight styles, and anthers 1-1.2 mm, whereas *A. canaliculatum* has thickish and opaque lateral pericarp, recurved styles, and anthers 0.5~0.8 mm long.

Our analysis showed that the *A. canaliculatum* - *A. lanceolatum* clade was the first diverged clade in *Alisma* (**Figure 6**). Jacobson and Hedrén (2007) also showed *A. lanceolatum* as sister to other *Alisma* species, and they argued that the allopolyploid origin of *A. lanceolatum* may have led to the hybrid ITS sequence produced by concerted evolution.

Group II: the North American clade of *A. subcordatum* and *A. triviale*: The two North American species *A. subcordatum* and *A. triviale* formed a clade with strong support (100% ML BS, 1 PP). But the two species are each unresolved. According to Björkqvist (1968), *A. triviale and A. subcordatum* both seem to be isolated from all other taxa of the genus with absolute sterility barriers, and both are distributed in New World (North America). *Alisma subcordatum* has a more southeastern distribution, whereas *A. triviale* has a more northern and western distribution in North America (Björkqvist, 1968).

With the two North American species each not forming a clade, it is worth mentioning that these two taxa are morphologically highly similar (Björkqvist, 1968). Haynes and Hellquist (2000) indicated that *A. triviale* and *A. subcordatum* were occasionally treated as varieties of a widespread *A. plantago-aquatica.* But they differ in ploidy levels with *A. triviale* as a polyploid (2*n* = 28) and *A. subcordatum* as a diploid (2*n* = 14) (Björkqvist, 1968). We thus speculate that *A. triviale* may be of allopolyploid origin in North America. We must test this hypothesis by employing nuclear genome data and conducting phylogenetic network analysis (Ferreira de Carvalho et al., 2019; Mendez-Reneau et al., 2023).

Group III: the clade of *A. gramineum* and *A. wahlenbergii*: Samuelsson (1931) first suggested that *A. wahlenbergii* was a relict endemic in the Baltic Sea area, but later regarded it as a newly formed taxon that had evolved as an ecotype of the morphologically similar *A. gramineum* at the shores of the Baltic Sea (Samuelsson, 1934). The latter hypothesis was also supported by Björkqvist (1968). Jacobson and Hedrén (2007) reported small genetic differences between *A. gramineum* and *A. wahlenbergii*, supporting the newly evolved hypothesis of *A. wahlenbergii*. Our result of comparative plastid genome and phylogenetic analyses is consistent with this hypothesis.

*Alisma gramineum* and *A. wahlenbergii* formed a clade with 100% support. The hypothesis that the Baltic endemic *A. wahlenbergii* was probably originated relatively recently from *A. gramineum* (Jacobson and Hedrén, 2007) may be supported by two aspects of evidence. First, both species live under water and their habitats obviously differ from those of the other congeneric species. *Alisma gramineum* distributes widely in Europe, Asia, and North America, and it is more adapted to submerged conditions than most other taxa of the genus. *Alisma wahlenbergii* has a more restricted distribution in Europe from Sweden, along the Finish coast of the Gulf of Bothnia and the Gulf of Finland, to the western part of Russia. Second, ecological environment determines that the morphology characters of the two species are very similar. *Alisma wahlenbergii* is similar to *A. gramineum* in many ways, including leaf shape, petal shape, anther shapes and size, style shape and length, achene shape. However, compared *A. gramineum*, *A. wahlenbergii* is more slender and smaller in most characteristics, both vegetatively and florally *(* Björkqvist, 1968).

Group IV: the clade of *A. plantago-aquatica* and *A. orientale*: *A. plantago-aquatica* from Eurasia and northern Africa and the eastern Asian *A. orientale* form a clade, yet the latter is nested within the widespread *A. plantago-aquatica*. The result hence supports treating *A. orientale* as a synonym of *A. plantago-aquatica*. But we will further test this hypothesis with the nuclear data.

The systematic relationship between the two taxa has been controversial. Based on the evidence of morphology, anatomy, pollen morphology and geographic distribution, Björkqvist (1968) and Zhang and Chen (1994) found that *A. orientale* from East and Central Asia was very uniform throughout the range and vegetatively similar to *A. plantago-aquatica*, but can be distinguished in floral characteristics, e.g. petal size, anther and style shape, stigma regions, shape of achenes, indicated that although there were interspecific and intraspecific variation in the two species, but most of these morphological variations were continuous and many were overlapping. The interspecific boundary between the two species seems blurred. The long-term cultivation and domestication of the well-known traditional medicine *Alismatis Rhizoma* in different areas in China may have also led to the formation of two different populations of “the *A. plantago-aquatica* complex”.

Rhizoma Alismatis is widely used in China, Japan, Malaysia and South Korea with over two thousand years of medicinal history. As the original plants of *Alismatis Rhizoma*, *A. plantago-aquatica* and *A. orientale* were listed in the latest edition of Chinese Pharmacopoeia (Pharmacopoeia of the People’s Republic of China, 2020), respectively. Although it had been cultivated for thousands of years in China, the botanical origins of the two species have caused much controversy due to the very little morphological distinctions. According to the *Flora of China* treatment (Wang et al., 2010), the distinguishing features between *A. plantago-aquatica* and *A. orientale* are few, primarily related to the length of styles, the margin of petals, and the regularity/irregularity of carpel arrangement. Additionally, these two species have significant geographic overlap in China (Wang and Li, 2002; Wu et al., 2008; Chen et al., 2009; Ma et al., 2015; Zhang et al., 2016). Based on morphological comparisons of the flowers and fruits, Liu et al. (2020) suggested that the differences between the two are very minimal. The identification results from DNA barcoding also reflected their similarities, with a single-base difference at position 247 out of 534 bases, and their homology exceeding 99.8%. Based on nuclear DNA (nrITS and *phyA*) and chloroplast DNA (*matK, ndhF, psbA-trnH* and *rbcL*) sequence data, Ito and Tanaka (2023) showed that the *A. planago-aquatica* complex including *A. orientale* was resolved to be monophyletic. They further suggest that *A. orientale* endemic to the Southeast Asian Massif may have originated via parapatric speciation from the progenitor species *A. planago-aquatica*, but the interspecific phylogenetic relationship was scarcely resolved.

Our results in this study challenge the species status of *A. orientale*, as it is nested within the widespread *A. planago-aquatica*. This result may also be related to the medicinal utilization history of nearly two thousand years and the very rare status of both species in the wild. Therefore, further analyses are needed to understand the phylogeographic pattern and species delimitation in this group by expanding the taxon sampling including both wild and cultivated in Asia especially in southeastern China and using nuclear genome analysis to further explore the phylogeny and delimitation of the two species. Such analyses will help reveal the origin, differentiation and domestication process of the important medicine “*Alismatis Rhizoma*” and provide an important reference for solving the taxonomic disputes for the two taxa.

Compared with traditional molecular phylogenetic studies based on several loci, the whole plastomes provide more informative sites for phylogenetic studies, which substantially improves the resolution of phylogenetic trees of some taxa at different taxonomic levels (Zhang et al., 2017b; Zhao et al., 2018). Indeed, compared with the previous molecular phylogenetic trees constructed from a few gene fragments (Jacobson and Hedrén, 2007; Ito and Tanaka, 2023), the support of the phylogenetic tree constructed based on the complete plastid genome data of *Alisma* in this study was much improved. But the plastomes still cannot fully resolve the phylogeny and species differentiation of *Alisma* due to allopolyploidy in the genus (Jacobson and Hedrén, 2007; Zhao et al., 2017). Therefore, future phylogenetic studies of *Alisma* need to be performed using nuclear genes, such as the Hyb-Seq approaches (Ma et al., 2021; Liu et al., 2023; Talavera et al., 2023) to unravel the allopolyploid speciation history of several polyploid species.

## 5 Conclusions

In this study, we sequenced and assembled the plastid genomes of eight species of the medicinally important genus *Alisma*, plus one species of *Echinodorus* and one species of *Luronium* of Alismataceae. The reported plastid genomes in *Alisma* are conserved, showing high levels of consistency and similarity in terms of gene content, order and structure. The plastid phylogenomic analyses have improved the phylogenetic resolution, and supported four main clades within *Alisma*. Our results support the tetraploid *A. lanceolatum* as the likely maternal parent of the hexaploid eastern Asian *A. canaliculatum*. However, plastid genomes still cannot resolve the complicated phylogenetic relationships and species discrimination especially within the North American clade and among polyploid species. Further studies are needed to test the extent of hybridization and allopolyploidy during the evolutionary diversification of *Alisma* using nuclear sequence data and a broader taxon sampling.

## Data Availability

The data presented in the study are deposited in the GenBank repository, accession number OR773541-OR773543, OR999289-OR999318, PP061022.
