# Supplementary material for: Comparative and phylogenetic analyses of plastid genomes of the medicinally important genus Alisma (Alismataceae)
Source: Front Plant Sci. 2024 Aug 20;15:1415253. doi: 10.3389/fpls.2024.1415253 (PMC11372848; doi:10.3389/fpls.2024.1415253)
Supplement: Supplementary file 1 [file DataSheet1.zip › Supplementary files/Supplementary Table 1.docx]

**Supplementary Table 1.** Specimens sampled for phylogenetic analysis including voucher information and geographic region.

| Genus | Species | Voucher information | Collection Date (YY/M/D) | Geographic origin | Accession number |
| --- | --- | --- | --- | --- | --- |
| *Alisma* | *A. canaliculatum* | L11-18786-US,Hatusima, S. | 1955.6.5 | Okinawa Island, Japan | OR999289 |
|  | *A. canaliculatum* | L46-2464-CDCM,Cheming Tan | 2017.7.30 | Jiangxi, China | OR999290 |
|  | *A. gramineum* | L20-1478-DBG:KHD,Melissa Islam | 2014.7.10 | Colorado, USA | OR999291 |
|  | *A. lanceolatum* | L12-12881-US,Koelz, W. N. | 1973.8.6 | Afghanistan | OR999292 |
|  | *A. lanceolatum* | L13-15217-US,Koelz, W. N. | 1940.5.7 | Chahar Mahaal and Bakhtiari, Iran | OR999293 |
|  | *A. lanceolatum* | L28-3069-GOET(Marc),Dersch | 1976 | Germany | OR999294 |
|  | *A. lanceolatum* | L29-s.n.-GOET(Marc),Miiller | 1994 | Germany | OR999295 |
|  | *A. lanceolatum* | L30-s.n.-GOET(Marc),Hofmann | 1972 | Slovenia | OR999296 |
|  | *A. lanceolatum* | L31-SOF73013-GOET(Marc),Lewejohann | 1973 | France | OR999297 |
|  | *A. lanceolatum* | L32-s.n.-GOET(Marc),Berger | 1958 | France | OR999298 |
|  | *A. orientale* | L15-4204-US,Koelz, W. N. | 1933.4.22 | Punjab, India | OR773541 |
|  | *A. orientale* | L44-FJ2023041001-CDCM,Shigui Mo | 2023.4.10 | Fujian, China | OR999299 |
|  | *A. plantago-aquatica* | L10-9319-US,Villaret, P. | 1947.8.24 | Vaud, Switzerland | OR999300 |
|  | *A. plantago-aquatica* | L18-203-US,X | 1964.7.29 | Federation; Leningrad, Russian | OR999301 |
|  | *A. plantago-aquatica* | L27-s.n.-GOET(Marc),vihodcesky | 1968 | Bulgaria | OR999302 |
|  | *A. plantago-aquatica* | L34-SC2023040301-CDCM,Wen Zheng | 2023.4.3 | Sichuan, China | OR999303 |
|  | *A. plantago-aquatica* | L35-SC2023040302-CDCM,Wen Zheng | 2023.4.3 | Sichuan, China | OR999304 |
|  | *A. plantago-aquatica* | L36-SC2023040303-CDCM,Wen Zheng | 2023.4.3 | Sichuan, China | OR999305 |
|  | *A. plantago-aquatica* | L37-SC2023040304-CDCM,Wen Zheng | 2023.4.3 | Sichuan, China | OR999306 |
|  | *A. plantago-aquatica* | L38-SC2023040305-CDCM,Wen Zheng | 2023.4.3 | Sichuan, China | OR999307 |
|  | *A. plantago-aquatica* | L39-SC2023041601-CDCM,Wen Zheng | 2023.4.16 | Sichuan, China | OR999308 |
|  | *A. plantago-aquatica* | L42-NMG2023041001-CDCM,Juan Yu | 2023.4.10 | Inner Mongolia, China | OR999309 |
|  | *A. plantago-aquatica* | L17-2993-US,Burger, W. C. | 1963.7.4 | Harar, Ethiopia | OR999315 |
|  | *A. subcordatum* | L1-2324-US,Fleming, P. | 1989.8.7 | Maryland, USA | OR999310 |
|  | *A. subcordatum* | L6-412-US,Van Neste, K. M. | 2015.7.1 | Virginia, USA | OR773542 |
|  | *A. subcordatum* | L9-5999-US,Hartley, T. G. | 1958.9.1 | Wisconsin, USA | OR999311 |
|  | *A. triviale* | L2-2281-US,Dennis, La Rea J. | 1960.8.4 | Oregon, USA | OR999312 |
|  | *A. triviale* | L4-413-US,Moss, E. H. | 1939.8.15 | Alberta, Canada | OR999313 |
|  | *A. triviale* | L5-12783-US,Kearney, T. H. | 1935.9.20 | Arizona, USA | OR999314 |
|  | *A. triviale* | L21-13366-DBG:KHD,Janet L. Wingate | 2019,7,26 | Colorado, USA | OR773543 |
|  | *A. wahlenbergii* | L16-4053-US,Juzepczuk, S.V. | 1955.8.19 | Petersburg, Russian | OR999316 |
| *Echinodorus* | *E. grisebachii* | L40-GX2023040401-CDCM,Xiaoqing Ning | 2023.4.4 | Guangxi, China | OR999317 |
|  | *E. grisebachii* | L43-JX2023041201-CDCM,Wei Qiu | 2023.4.12 | Jiangxi, China | PP061022 |
| *Luronium* | *L. natans* | L48-30TUN5333-US,Cirujano, S. | 2001.9.5 | Castillay León, Spain | OR999318 |
| *Sagittaria* | *S. graminea* | / | / | China | NC_067603 |
|  | *S. trifolia* | / | / | Xinjiang, China | NC_044119 |
|  | *S. lichuanensis* | / | / | Wuhan, China | NC_029815 |
| *Caldesia* | *C. grandis* | / | / | Hunan, China | NC_045925 |
|  | *C. parnassifolia* | / | / | Hunan, China | NC_045926 |
| *Butomus* | *B. umbellatus* | / | / | Xinjiang, China | NC_051949 |
